# Association between Proinflammatory Cytokines and Anxiety and Depression Symptoms in Rheumatoid Arthritis Patients: A Cross-sectional Study

**DOI:** 10.2174/17450179-v19-e230510-2022-34

**Published:** 2023-06-22

**Authors:** Faisal Parlindungan, Rudy Hidayat, Anna Ariane, Hamzah Shatri

**Affiliations:** 1 Department of Internal Medicine, Division of Rheumatology, Faculty of Medicine, Universitas Indonesia, Dr. Cipto Mangunkusumo National General Hospital, Jakarta, Indonesia; 2 Department of Internal Medicine, Division of Psychosomatic and Palliative, Faculty of Medicine, Universitas Indonesia, Dr. Cipto Mangunkusumo National General Hospital, Jakarta, Indonesia

**Keywords:** Anxiety, Depression, Arthritis rheumatoid, Inflammation, Interleukin-6, Interleukin-17, Tumor necrosis factor-alpha

## Abstract

**Background:**

Rheumatoid arthritis (RA) patients have a greater prevalence of anxiety and depression. Proinflammatory cytokines are elevated in RA. We aim to evaluate the association between systemic inflammation in RA and anxiety and depression.

**Methods:**

There were 31 RA patients, 16 with active disease activity and 15 in remission state; they were assessed using the Hospital Anxiety and Depression Scale and for RA disease activity using Disease Activity Score of 28 joints (DAS28) – CRP (C-reactive protein). Serum proinflammatory cytokines were measured, including interleukin (IL)-6, IL-17, and Tumour Necrosis Factor-alpha (TNF-α).

**Results:**

Among 31 patients, ten patients showed anxiety symptoms, 19 patients showed depression symptoms, and two displayed mixed symptoms. Serum TNF-α levels were significantly higher in active disease than in the remission group (p-value 0.006). There was no association or correlation between proinflammatory cytokines to anxiety and depression symptoms in the active disease and remission groups.

**Conclusion:**

This suggests that other factors besides disease activity and state of systemic inflammation may cause anxiety and depression in RA patients.

## 1 INTRODUCTION

Rheumatoid arthritis (RA) is a chronic autoimmune inflammatory condition that causes inflammation and joint damage. It can also display extra-articular manifestations, which affect major organs of the body [1]. As a result, RA patients have a greater prevalence of physical disability and psychiatric comorbidity [2]. The most common psychiatric conditions related to RA are depression and anxiety [3], where 14% to 48% of RA patients have depressive symptoms [4], and about 16% to 40% of RA patients experience anxiety symptoms [5].

Anxiety and depression symptoms may occur from multifactorial causes [2, 3], but systemic inflammation may also play a certain role in the immune-brain pathway [6]. In addition, in RA patients, there might be an overlap between depression and anxiety [3]. This may increase the disease activity, a reduced response to RA symptom treatment, poor medication adherence, increased health service utilization, and a decreased likelihood of achieving RA symptom remission, disability, and mortality [1, 2, 6-8].

The pathogenesis of RA is associated with an increase in biomarkers of inflammation, including C-reactive protein (CRP), interleukin 1 (IL-1), interleukin 6 (IL-6), and Tumour Necrosis Factor-alpha (TNF-α) [4]. Proinflammatory cytokines contribute to psychoneuroimmunological disturbances [6, 9-11]. An increase of IL6, IL-1, IL-17, and TNFα in peripheral blood related to anxiety and depression compared to healthy controls [9, 12, 13]. These released cytokines are transported to the central nervous system (CNS) through the blood-brain barrier or circumventricular organs [9]. It affects neurotransmitter and hormone secretion in the amygdala and hippocampus as an area of anxiety control, the center of pain, and the hypothalamic-pituitary-adrenal (HPA) axis as the component of stress regulation during physical and psychological stress. Thus, neurotransmitter and hormone imbalances, such as serotonin, dopamine, noradrenaline, and cortisol, are known to be related to anxiety and depression [9, 12, 14-18].

It is still unclear whether systemic inflammation might induce a psychological functioning disturbance in RA. This study aims to associate systemic inflammation, disease activity, anxiety, and depression in RA patients.

## 2 MATERIALS AND METHODS

### 2.1 Study Population

This was a cross-sectional study conducted at the outpatient rheumatology clinic of Cipto Mangunkusumo General Hospital (Jakarta, Indonesia) consecutively from April-May in 2019. The inclusion criteria were premenopausal females with RA, diagnosed according to the 2010 European League Against Rheumatism/American College of Rheumatology (EULAR/ACR) classification criteria for RA who consented to join this study. We excluded all subjects consuming steroids equivalent to prednisone > 7.5mg per day for three consecutive months, patients with bone metabolism disorder as recorded in the medical record (such as hyperparathyroidism, osteomalacia, osteogenesis imperfecta, Paget's disease), patients having an autoimmune disease other than RA (such as lupus, psoriatic arthritis, systemic sclerosis), patients with medications which affect bone metabolism (such as hormonal therapy, anti-epileptic drugs, bisphosphonate, anticoagulant, diuretics), patients with end-stage chronic kidney disease with glomerular filtration rate <15 mL/minute, patients with chronic liver disease (such as liver cirrhosis, hepatoma), and any acute infectious conditions (such as pneumonia, acute fever).

### 2.2 Disease Activity Score for 28 joints - C-reactive Protein (DAS28-CRP)

We evaluated the disease activity in patients with RA using the Disease Activity Score for 28 joints (DAS28). We calculated the score after four variables: number of painful joints, number of swollen joints, C-reactive protein (CRP), and patient's global assessment (PGA) of disease activity on a 100-mm visual analog scale (VAS). We defined a DAS28 value of >5.1 indicating high disease activity, 3.2 <DAS28 ≤5.1, and DAS28 ≤3.2 as moderate and low disease activity, and we considered patients to be in the remission phase if DAS28 was <2.6 [19].

### 2.3 Hospital Anxiety and Depression Scale (HADS)

Anxiety and depression status were measured using the Hospital Anxiety and Depression Scale (HADS) for the setting of a hospital medical outpatient clinic. We subdivided the HADS into two subscales of 7 items. One subscale measures depression, and one measures anxiety. The scores for each scale can be between 0 and 21. Scores ranging from 8 to 10 are considered mild, 11 to 14 moderate, and 15 to 21 severe. For this study, ≥8 was used as the point indicating either anxiety or depression [20]. The Indonesian version of HADS has been proven as a valid and reliable questionnaire and has been used in several previous studies with Indonesian patients [21, 22].

### 2.4 Cytokine Analysis

We collected the blood samples from patients in the morning (between 07.30 and 10.00 a.m.) after 8 hours of fasting due to diurnal variability of CTx serum on the same day they completed the self-report questionnaire. Serum specimens were stored frozen (-20^0^C) until the time of analysis. IL-6, IL-17, and TNF-α were analyzed by quantitative sandwich enzyme immunoassay (R&D systems, UK). Each cytokine assay was performed on one 96-well plate, and the optical density of each was measured using a microplate reader set to 450 nm. The measuring range for each assay was IL-6, 3.1 -300 pg/mL; IL-17, 31.2 – 2,000 pg/mL; and TNF-α, 15.6 – 1,000 pg/mL.

### 2.5 Statistical Analysis

Collected data were analyzed using SPSS 20.0 (SPSS INC., Chicago, IL, USA) for Mac. Descriptive data were presented in the form of the mean (Standard Deviation) when they were normally distributed or median (min-max) when they were not normally distributed. Statistical analyses for the differences in cytokine levels between active and remission and within the disease activity with and without anxiety and depression were compared using the Mann–Whitney U-test or the independent t-test. Spearman's rank correlation explored the correlations between proinflammatory cytokines, disease activity, anxiety and depression. All statistical tests were 2-sided, and P values less than 0.05 were significant.

## 3 RESULTS

About 31 RA patients fulfilled the criteria, as shown in Table **1**. All subjects were female, with a mean disease duration of 5.6±3.56 years. The most common DMARD (Disease-Modifying Antirheumatic Drug) medication was methotrexate as the drug of choice for RA. The majority of the patient received methylprednisolone equal to 4 mg 22 (71.0%), while 5 (12.9%) were not using methylprednisolone. There were 15 patients (48.4%) in a remission state, while 16 patients experienced active disease activity with high, moderate, and low disease activity.

Patients were divided into active disease and remission groups matched by age and gender as seen in Table **2**. The TNF-α level was significantly higher in the active disease group than in the remission group (10.81 pg/ml *vs*. 9.82 pg/ml; p-value 0.006). IL-6 level was higher in the active disease group than in the remission group but statistically insignificant (18.26 pg/ml *vs*. 3.83 pg/ml; p-value 0.102). There was no difference in IL-17 levels between the active disease and remission groups (10.38 pg/ml *vs*. 10.51 pg/ml; p-value 0.889).

Subjects with anxiety symptoms were higher in the active disease group than the remission group (45.1% *vs*. 41.9%), and we found no associations between anxiety symptoms and disease activity (p-value 0.797). All subjects in both the active disease group and remission group had depression.

We did a subgroup analysis of all the subjects, including those with an overlap between anxiety and depression. As shown in Table **3**, in the active disease group, the TNF-α level was lower in the subjects experiencing anxiety symptoms but statistically insignificant (10.72 pg/ml *vs*. 11.17 pg/ml, p-value 0.383). Compared to the remission group, TNF-α was significantly higher in patients with anxiety in the active disease group (11.67 pg/ml *vs*. 9.61 pg/ml, p-value 0.003). In the active disease group, IL-6 level was lower in the subjects experiencing anxiety symptoms but statistically insignificant (1.00 pg/ml *vs*. 2.81 pg/ml, p-value 0.573). Compared to the remission group, the IL-6 level was lower in the active disease group but statistically insignificant (1.00 pg/ml *vs*. 3.99 pg/ml, p-value 0.076). There was no difference in IL-17 levels in the anxiety and non-anxiety group (10.58 pg/ml *vs*. 10.78 pg/ml, p-value 0.910), and there was also no difference in IL-17 levels in the active disease and remission group (10.58 pg/ml *vs*. 10.42, p-value 0.995).

As shown in Table **4**, compared to the remission group, there was a significantly higher level of TNF-α in the depression group (11.45 pg/ml *vs*. 9.69 pg/ml, p-value 0.006). Compared to the remission group, the TNF-α level was higher in the active disease group but statistically insignificant (18.27 pg/ml *vs*. 3.84 pg/ml, p-value 0.085). Compared to the remission group, there was no difference in IL-6 level (10.55 pg/ml *vs*. 10.47 pg/ml, p-value 0.920).

As shown in Table **5**, there was no correlation between TNF-α, IL-6, and IL-17 to anxiety and depression symptoms, either in the active disease group or the remission group.

## 4 DISCUSSION

### 4.1 Proinflammatory Cytokines and Disease Activity

There are many cytokines in RA pathogenesis, but TNF, IL-6, IL-1, IL-17, and GM-CSF are the main cytokine of RA [23-25]. In our study, we found a significantly higher level of TNF-α in the active disease group compared to the remission group, and there were no differences in both groups. Our study yielded different results than similar studies by Li *et al.* [2] and Liu *et al.* [12], where IL-6, IL-17, and TNF-α were higher in RA patients than in healthy subjects. Our study compared RA patients with active disease to patients in remission, not to a healthy subject.

In our study, there were significantly higher TNF-α levels in the active disease group than in the remission group. Physiologically, TNF is the main cytokine in the inflammatory process; it recruits inflammatory immune cells and promotes tissue destruction. In pathogenic function, it plays an essential role in inducing chronic uncontrolled production of proinflammatory mediators by many cells and inhibiting regulatory T cells' function. This wide variety of effector functions of TNF is relevant to RA pathogenesis and the progressivity of the disease [23]. In a meta-analysis study by Bek *et al.*, after the blockade of TNF-α using anti-TNF treatment, only 30–40% of the subject had no or insufficient response, which made patients remain still in high disease activity [26].

### 4.2 Disease Activity and Psychological Symptoms

In a retrospective cohort study by Marrie *et al.*, annual depression and anxiety disorder incidence rates were significantly higher in RA subjects than in the general population [27]. In our study that included only RA patients, 31% and 47% experienced anxiety in active and remission groups, respectively, but there was no association between disease activity and anxiety symptoms (p-value 0.379). It was consistent with previous studies where the estimated prevalence of anxiety symptoms in RA was 14-40%, 11.2% of patients were diagnosed with panic attacks, and 5.6% were diagnosed with generalized anxiety disorder [3, 8, 28].

In this study, 81% and 53% of the patients had depression symptoms in active and remission groups. There was no association between disease activity and anxiety symptoms (p-value 0.097). This number was higher than the previous meta-analysis study by Matcham *et al.* The estimated prevalence of individuals with RA who experienced depressive symptoms who were evaluated using HADS was 34.2%, and the prevalence of major depressive disorders according to the Diagnostic and Statistical Manual of Mental Disorders criteria was 16.8% [29].

### 4.3 Proinflammatory Cytokines and Depression Symptoms

In our study, the TNF-α level in the depression group was higher in the active disease group than in the remission group. There was no different level in the active disease group between the depression and non-depression groups. A meta-analysis study by Dowlati *et al.* showed that TNF-α level was higher in depressed subjects than in non-depressed subjects, but this study compared RA and other inflammatory diseases to healthy subjects [30]. In our study, there was no correlation between TNF-α and depression symptoms. This result was consistent with similar studies by Li *et al.* [2], Liu *et al.* [12], and El-Tantawy *et al.* [31], which stated that TNF-α does not correlate with depression symptoms.

In our study, IL-6 and IL-17 levels in the depression group had no differences in the active disease group compared to the remission group. There was no different level in the depression group in the active disease group than in the non-depression group. This result was inconsistent with a meta-analysis study by Dowlati *et al.*, where IL-6 and IL-17 levels were higher in the depressed subject than in the non-depressed subject; this study compared RA and other inflammatory diseases to a healthy subject [30]. Our study also showed no correlation between IL-6 and IL-17 to depression symptoms. This result was consistent with Liu *et al.* [12], which stated that there was no significant difference and no correlation between IL-16 and IL-17 to depression symptoms compared to the healthy subjects. However, Figueiredo-Braga *et al.* [32] and Li *et al.* [2] stated that IL-16 and IL-17 contribute to depression symptoms.

### 4.4 Proinflammatory Cytokines and Anxiety Symptoms

In our study, the TNF-α level in the anxiety symptom group in the active disease group was higher than in the remission group. In the active disease group, the TNF-α level was lower in the anxiety group compared to the non-anxiety group. There was also no correlation between TNF-α and depression symptoms. This result was consistent with a similar study by Liu *et al.* [12], where there was no different level of TNF-α between patients with and without anxiety symptoms. There was also no correlation between TNF-α level and anxiety symptoms in our study. It was also consistent with both Liu *et al.* [12] and Li *et al.* [2], which stated no correlation between TNF-α levels and anxiety symptoms.

In our study, IL-6 had no different level in the active disease group compared to the remission group. IL-6 was lower in the anxiety group than in the non-anxiety group in the active disease group but statistically insignificant, consistent with similar studies by Liu *et al.* [12] and Li *et al.*, where there was no different level of IL-6 between patients with and without anxiety symptoms. There was no correlation between IL-6 levels and anxiety symptoms in our study. It was also consistent with Liu *et al.* [12], Li *et al.* [2] and El-Tantawy *et al.* [31], which stated there was no correlation between TNF-α levels and anxiety symptoms.

In our study, IL-17 had a different level in the active disease group compared to the remission group. IL-17 had no different level in the anxiety group than the non-anxiety group in the active disease group, but statistically insignificant. Inconsistent with a similar study by Liu *et al.* [12], the serum IL-17 levels in RA patients with anxiety were significantly higher than those without anxiety. There was no correlation between IL-17 levels and anxiety symptoms in our study. It was also inconsistent with Liu *et al.* [12], which stated that the IL-17 level was positively correlated to anxiety symptoms.

### 4.5 Systemic Inflammation and Psychoneuroimm-unological Disturbances

Systemic inflammation may contribute to psychoneuroimm-unological disturbances [6, 9-11]. This hypothesis was supported by meta-analyses showing that cytokine concentrations, including IL6, IL1β, IL17, and TNFα, were raised in the peripheral blood of patients with depression and anxiety compared with healthy controls [9, 12, 13]. These cytokines may affect CNS through several mechanisms. First, in terms of the humoral route circulating cytokines (TNF-α and IL-17), they can be actively transported through the blood-brain barrier endothelium mediators and can activate the blood-brain barrier endothelium, or inflammatory molecules which might access the CNS through the circumventricular organs [9]. For example, IL-17 may cross the blood-brain barrier and affect neurotransmission in the amygdala and hippocampus, which are considered areas for anxiety control [12]. Second, in terms of a neural route of communication, the best-described model is the inflammatory reflex, which transmits ascending afferent information about peripheral immune status to the CNS and descending efferent information that modulates peripheral immune responses, for example, in pain response modulated by IL-6 [9, 12, 14]. The central action of cytokines may affect the hypothalamic-pituitary-adrenal (HPA) axis disturbance [15]. HPA is a central component of the stress system, responsible for the balance of physical and physiological responses to stressful situations and is a physiological self-regulatory system responsive to negative GC feedback [16]. Proinflammatory cytokines (IL-1, IL-6, TNF-α) in chronic disease may cause HPA axis hyperactivity by disturbing the negative feedback inhibition of circulating cortisol, which creates a condition of hypercortisolemia. Cortisol is a stress hormone related to fatigue, depression, and anxiety [15, 17]. Cytokines (IL-6, IL-17, and TNF-α) may also affect the secretion of neurotransmitters, such as serotonin, dopamine, and noradrenaline. The imbalance of those neurotransmitters was correlated with motivational, physical, and cognitive fatigue. Those neurotransmitters were also known to be related to anxiety and depression [18].

Besides RA, IL-6, IL-17, and TNF-α also increase in malignancy conditions, including colorectal cancer, breast cancer, and pancreatic cancer, which implicates the pathogenesis of anxiety and depression [33-38]. Furthermore, a study by Du *et al.* [39] stated that IL-6 and TNF-α may become a biomarker and novel targeted therapeutic interventions for depression in lung cancer patients. Hence, it can also be applied to RA patients that received IL-6 and TNF inhibitors. Further research should be done to evaluate improvement in mood disturbances in RA patients receiving treatment.

However, systemic inflammation may not be the only factor contributing to depression symptoms in patients with RA. RA patients with depression have impaired coping responses to pain, fatigue, and disability, leading to reduced physical exercise and engagement in social interaction. Subsequently, the decline in physical health and function increases emotional distress, frustration, and depression [1, 40]. Furthermore, a cross-over between pain, fatigue, and depression is likely to have a role in the higher rates of depression and cause exacerbated depression [9]. In addition, genes encoding cytokines are associated with an increased or reduced cytokine release. Moreover, abnormal cytokine allele gene variation may reduce the responsiveness of antidepressant therapy [41].

External factor also contributes to the symptoms. For example, low socioeconomic statuses, such as income, education, occupation, race/ethnicity, and neighborhood conditions are associated with depression in RA. Patient characteristics, such as female gender, younger age, race/ethnicity, poor coping mechanisms, and decreased social support, are also associated with depression in RA [6, 42]. In another multicentre cross-sectional study by Katchamart *et al.*, functional disability, those who were married, had cognitive impairment, had been diagnosed with RA for <10 years, and had higher pain scores significantly associated with anxiety [43]. In addition to this, human bonding plays a role in mood disorder in RA patients, the more secure the attachment style to their partner, the less anxiety and avoidance behavior sought for help and comfort, and vice versa. However, gender affects this attachment style. Finally, diets such as an anti-inflammatory diet in rheumatoid arthritis and higher dietary inflammatory index food, macronutrients, and probiotics give a better outcome in regulating cytokine release, and disease progression correlated with depression [44].

## 5 LIMITATIONS

The limitation of this study was that this was a cross-sectional study that could not explain causal and effect relations. Second, our sample size was relatively small, and all of the RA patients in our clinic during the study course were female, which might have caused a sampling bias that was not representative of the general population. However, RA predominantly affects the female population. Moreover, we did not evaluate the healthy subject as we wanted to compare the incidence of depressive and anxiety symptoms among RA patients and its correlation to their different levels of cytokines and disease activity profile. Third, we did not evaluate socioeconomic factors, which could become cofounders and biased on the depression symptoms. Lastly, we used HADS scoring to evaluate anxiety and depression symptoms, a screening tool to assess psychological problems in the setting of the outpatient clinic for chronically ill patients of RSUPN Cipto Mangunkusumo and other hospitals in Indonesia, which does not diagnose the major depressive disorder or generalized anxiety disorder.

## CONCLUSION

In conclusion, Serum TNF-α level was elevated in RA patients with active disease. There was no association between proinflammatory cytokines and anxiety and depression symptoms between RA patients with active disease and those in remission.

## Figures and Tables

**Table 1:** Subject characteristic.

**Characteristics**	**Population** **n=38**
Age (in years, mean ± SD)	37.61 ± 6.98
Duration of RA (in years, median, minimum-maximum)	5.6 ± 3.56
DMARD used (n, %) MTX monotherapy MTX combination therapy DMARD other than MTX	18 (58.1%) 8 (25.8%) 5 (16.1%)
Glucocorticoid use (n, %) Not using Methylprednisolone < 4mg or equivalent Methylprednisolone =4mg or equivalent	5 (16.1%) 4 (12.9%) 22 (71.0%)
DAS28 CRP (n, %) Remission Low Moderate High	15 (48.4%) 4 (12.9%) 11 (35.5%) 1 (3.2%)
TNF-*α* (pg/mL, mean ± SD)	10.59 ± 1.89
IL-6 (pg/mL, median, minimum-maximum)	3.53 (1.56-119.74)
IL-17 (pg/mL, mean ± SD)	10.51 ± 0.63

**Abbreviations:** DMARD: Disease-Modifying Antirheumatic Drug; MTX: Methotrexate, TNF-á: Tumor Necrosis Factor-α; IL: Interleukin; DAS28-CRP: Disease Activity Score-28-C-Reactive Protein SD: Standard Deviation

**Table 2:** Group of disease activity matched by age and gender.

**-**	**Total**	**Active Disease** **(n=16)**	**Remission** **(n=15)**	**p-value**
**Pro-Inflammatory Cytokine^#^**				
TNF-α (pg/ml), Median (Min-Max)	31	10,81 (9,22-17,67)	9,82 (7-12,16)	0.006*
IL-6 (pg/ml), Mean (SD)	31	18,26 (0,70; 35,82)	3,83 (1,99; 5,68)	0.102
IL-17 (pg/ml), Median (Min-Max)	31	10,38 (9,74-11,85)	10,51 (9,48-11,34)	0.889
Anxiety*				
Anxiety (%)	27	14 (45.1%)	13 (41.9%)	0.797
Non-Anxiety (%)	4	2 (6.5%)	2 (6.5%)	
Depression*				
Depression (%)	31	16 (100%)	15 (100%)	
Non-Depression (%)	0	0 (0%)	0 (0%)	

**Note:** ^#^= Mann-Whitney test; *= Chi-square test; TNF-á: Tumor Necrosis Factor-α; IL: Interleukin; HADS: Hospital Anxiety and Depression Scale; SD: Standard Deviation; *p > 0.05: non-significant; p < 0.05: significant; **p < 0.001: highly significant

**Table 3:** Comparison of pro-inflammatory cytokines, anxiety symptoms, and disease activity.

**Proinflammatory Cytokines**	**Active Disease Group (n=16)**		-	**Remission Group (n=15)**	
	**Anxiety** **(n= 14)**	**Non-Anxiety** **(n= 2)**	**p-value**	**Anxiety** **(n= 13)**	**p-value**
TNF-α (pg/ml), Mean (SD)	11.67 (9.67-17.67)	10.21 (9.07-11.36	0.809	9.61 (7.00-12.16)	0.003*
IL-6 (pg/ml), Median (Min-Max)	1.00 (1.00-1.00)	2.81 (2.09-3.53)	0.573	3.99 (1.56-14.43)	0.076
IL-17 (pg/ml), Mean (SD)	10.58 (0.66)	10.78 (0.75)	0.910	10.42 (0.65)	0.995

**Note:** TNF-á: Tumor Necrosis Factor-α; IL: Interleukin; *p > 0.05: non-significant; p < 0.05: significant; **p < 0.001: highly significant

**Table 4:** Comparison of pro-inflammatory cytokines, depression symptoms, and disease activity.

**Proinflammatory Cytokines**	**Active Disease Group (n= 16)**	**Remission Group (n=15)**	
	**Depression** **(n= 16)**	**Depression** **(n= 8)**	**p-value**
TNF-α (pg/ml), Mean (SD)	11.45 (9.22-17.67)	9.69 (7.00-12.16)	0.006*
IL-6 (pg/ml), Median (Min-Max)	18.27 (2.09-199.74)	3.84 (1.56-14.43)	0.085
IL-17 (pg/ml), Mean (SD)	10.55 (9.74-11.85)	10.47 (9.48-11.34)	0.920

**Note:** ^a^: Independent t-test; ^b^: Mann-Whitney; TNF-á: Tumor Necrosis Factor-α; IL: Interleukin; *p > 0.05: non-significant; p < 0.05: significant; **p < 0.001: highly significant

**Table 5:** Correlation between anxiety, depression, and pro-inflammatory cytokines in RA and controls.

**-**	**Active Disease (n=16)**					**Remission (n= 15)**					
**-**	**Anxiety**			**Depression**			**Anxiety**			**Depression**	
**-**	**r†**	**p-value**		**r†**	**p-value**		**r†**	**p-value**		**r†**	**p-value**
**TNF-α**	-0.36	0.894		-0.061	0.822		-0.880	0.755		0.162	0.565
**IL-6**	-0.005	0.987		0.113	0.676		0.124	0.659		0.097	0.732
**IL-17**	0.068	0.802		0.252	0.346		-0.199	0.477		-0.058	0,837

**Note:** †: Spearman’s rho coefficient; TNF-á: Tumor Necrosis Factor-α; IL: Interleukin; *p < 0.05: correlated, p > 0.05: no correlation

## Data Availability

All data and materials in the repository were the authors’ own data.

## LIST OF ABBREVIATIONS

DAS28: = Disease Activity Score of 28 joints
RA: = Rheumatoid Arthritis
CRP: = C-reactive Protein
IL: = Interleukin
TNF- α: = Tumour Necrosis Factor-alpha
GM-CSF: = Granulocyte-macrophage Colony-stimulating Factor
HPA: = Hypothalamic-Pituitary-Adrenal
EULAR/ACR: = European League Against Rheumatism/American College of Rheumatology
PGA: = Patient’s Global Assessment
VAS: = Visual Analog Scale
HADS: = Hospital Anxiety and Depression Scale
DMARD: = Disease-Modifying Antirheumatic Drug.
